# The effects of response inhibition training following binge memory retrieval in young adults binge eaters: a randomised-controlled experimental study

**DOI:** 10.1038/s41598-022-12173-w

**Published:** 2022-06-03

**Authors:** Ravi K. Das, Emma A. Cawley, Louise Simeonov, Giulia Piazza, Ulrike Schmidt, Reinout W. H. J. Wiers, Sunjeev K. Kamboj

**Affiliations:** 1 Clinical, Educational and Health Psychology, University College London, Gower Street, London, WC1E 6BT UK; 2 Institute of Psychiatry, Psychology and Neuroscience, King’s College London, 16 De Crespigny Park, London, SE5 8AF UK; 3 Faculty of Social and Behavioural Sciences, University of Amsterdam, Postbus 15916, 1001 NK Amsterdam, The Netherlands

**Keywords:** Human behaviour, Nutrition disorders, Diseases, Addiction

## Abstract

Binge eating is increasingly prevalent among adolescents and young adults and can have a lasting harmful impact on mental and physical health. Mechanistic insights suggest that aberrant reward-learning and biased cognitive processing may be involved in the aetiology of binge eating. We therefore investigated whether recently developed approaches to catalyse brief interventions by putatively updating maladaptive memory could also boost the effects of cognitive bias modification training on binge eating behaviour. A non-treatment-seeking sample of 90 binge eating young adults were evenly randomised to undergo either selective food response inhibition training, or sham training following binge memory reactivation. A third group received training without binge memory reactivation. Laboratory measures of reactivity and biased responses to food cues were assessed pre-post intervention and bingeing behaviour and disordered eating assessed up to 9 months post-intervention. The protocol was pre-registered at https://osf.io/82c4r/. We found limited evidence of premorbid biased processing in lab-assessed measures of cognitive biases to self-selected images of typical binge foods. Accordingly, there was little evidence of CBM reducing these biases and this was not boosted by prior ‘reactivation’ of binge food reward memories. No group differences were observed on long-term bingeing behaviour, caloric consumption or disordered eating symptomatology. These findings align with recent studies showing limited impact of selective inhibition training on binge eating and do not permit conclusions regarding the utility of retrieval-dependent memory ‘update’ mechanisms as a treatment catalyst for response inhibition training.

## Introduction

Binge Eating disorder (BED) is on the rise in young adults^1^. BED is notable due to its high prevalence across genders (estimated 1–4%^2^). It can be disabling due to its high comorbidity with anxiety^3^ and depression^4^ and it is statistically associated with physical health risk factors^5^ such as excess adiposity^6^ diabetes^7^ and metabolic syndrome^8^. Current therapies for BED are typically CBT or combined pharmacotherapy-based, and may effectively reduce binge frequency^9^. However, these therapies have high long-term relapse rates^10^, with only a minority achieving remission following treatment^11^. BED thus constitutes an enormous financial and healthcare burden within the EU^12^. Phenomenological similarities to, and high comorbidity with, substance use disorders suggest that binge/over-eating may share some underlying neurobiological and psychological aetiology with addiction^13–16^. Indeed the framing of these disorders as types of ‘food addiction’^13^ although controversial, is increasingly prevalent^17,18^. While ‘food addiction’ has been criticised for providing an incomplete account of binge eating (see Refs.^19,20^, for a discussion) with alternative (though not incompatible) mechanisms such as negative affect, dietary restraint^21^ and beliefs also playing a role^22^, most authors acknowledge an important contributory role of reward and motivational mechanisms in predisposing to binge eating in the modern food environment. As such, insights into maladaptive reward processes from the field of addiction (and novel strategies targeting these) might also be usefully applied in binge eating, offering new opportunities for prevention and intervention.

Aberrant reward processing is thought to be a core ‘transdiagnostic’ mechanism in addiction and binge eating aetiology^23–25^. Under this model, heightened reward responses to binge food-related ‘cues’ trigger automatic food-seeking (approach), ‘hedonic hunger’, preoccupation with food^16^, craving and maladaptive consumption behaviour. Indeed, patterns of craving and eating for reward enhancement distinguish binge-eating individuals from weight-equivalent healthy control populations^23^.

These reward responses are thought to be learned, rather than innate. Binged-on foods are almost universally ‘highly palatable foods’ (HPFs; highly processed foods with high caloric density and high fat/sugar macronutrient profile^26^), which produce reward and hedonic responses via sharp increases in ventral striatal dopamine^27^, and endorphin signalling^17^, but comparatively low satiety. These properties promote *overconsumption* (far exceeding homeostatic requirements) and support associative learning about sensory cues (tastes, textures, smells and visual qualities) that predict HPF reward, conferring high ‘addictive potential’ to HPFs^28,29^ and imbuing these cues with high salience and incentive properties^30^. HPF cues can thus elicit attentional capture^31,32^ and automatic motor ‘approach’ responses when encountered^33–35^, in a similar manner to drug-related stimuli in substance-use disorders (SUDs)^36^. Theoretically, automatic approach and motivational processes elicited by HPF cues require opponent top-down inhibition of responses to reward cues to over-ride impulsive–compulsive consumption, but this is thought to be impaired in binge eating^37^. High impulsivity and reduced inhibitory control capacity may thus conspire to support binge eating behaviour^38^.

Response inhibition training (RIT), a sub-type of ‘cognitive bias modification’(CBM) , broadly aims to retrain automatic behavioural biases to eating cues, and might improve outcomes in binge-eating individuals^23,39^. Response inhibition training may improve food-specific inhibitory control and reduce cue-induced motor activation by overriding prepotent ‘cue → go’ tendencies^40^ with inhibitory ‘cue → *no-go*’ responses. It is typically implemented via a ‘Go/No-go’ task, in which food cues (e.g. HPF images) are consistently paired with ‘no-go’ responses^41^. This has been found to reduce chocolate craving^42^, weight and overeating in ‘normal weight’, ‘overweight’^43^ and ‘obese’ individuals (primary researchers’ own terms)^44^. It has been suggested to be particularly effective in those with high BMI, who desire to lose weight, supporting a role of prepotent action biases in *excessive* consumption, and of motivation in ameliorating these^41^. However, most evidence from single-session laboratory studies on RIT is in healthy or ‘overweight controls’ and effects may be modest in disordered eating populations^45^ particularly when clinical endpoints are used^45,46^. In these populations, the comparatively brief nature of RIT may be insufficient to counteract the years of maladaptive learning that has ingrained ‘go’ biases to HPFs.

Recent successes in the fear and addiction literature suggest it may be possible to surmount this short-lived efficacy by using maladaptive learning reminders to catalyse brief learning-based interventions. The effects of these reminders are typically attributed to reconsolidation-update; a ‘housekeeping’ mechanism for memory maintenance^47^. Reconsolidation putatively serves to selectively strengthen, weaken or update memories by incorporating new information, dependent on the prediction of predict salient outcomes by the memory trace. The reconsolidation process follows (and requires) the retrieval-dependent destabilisation of memories. If novel cue-response associations are presented or trained while memories are labile, or if reconsolidation is pharmacologically halted^48^, memories may putatively be either modified^49,50^ or weakened. Since food ‘go’ biases are learned (and therefore stored in memory ), if the reward associations between binge food cues and HPF reward can be destabilised, subsequent RIT may directly *update* cue-response relationships, greatly catalysing its efficacy. This approach has demonstrated lasting efficacy (at least 9 months) when applied to experimental ‘ultra-brief’ behavioural treatment modalities (exposure^51^, counterconditioning^50^, cognitive reappraisal^52^).

We therefore sought to examine whether single-session RIT to binge food cues could reduce subsequent response biases to HPF, binge frequency and symptomatology in a group of young-adult, sub-clinical binge eating individuals and whether this effect could be similarly boosted by a ‘reminder’ of maladaptive learning prior to training, consistent with a reconsolidation-update mechanism. This population was targeted as they displayed clear bingeing behaviour, but were not currently receiving treatment, with which the current experimental approach might interfere, conveying higher risk of iatrogenic harm. They also represent an important target group in their own right, in which low-intensity interventions such as this might play an important preventative role.

## Methods

### Participants and design

The study design and analysis was pre-registered on the Open Science Framework on 02/03/2018 (https://osf.io/82c4r/) and ISRCTN on 23/01/2019 (https://doi.org/10.1186/ISRCTN13262256). Young adults (aged 18–25) with sub-clinical bingeing behaviour (≥ 1 binge/month and Binge Eating Scale (BES) score > 17) were assigned using block randomisation to three groups in a single-blind, randomised experimental study: Groups were: Binge Memory reactivation + Response Inhibition Training (*BMR* + *RIT*), No memory reactivation + RIT (*NR* + *RIT*), or BMR + ‘sham’ RIT (BMR + sham), all N = 30 (total randomised N = 90). These groups allowed us to assess effects of RIT *per se* and via putative reconsolidation-update. Full inclusion, exclusion and randomisation protocols and power calculation are detailed in the Supplementary Information*.* All procedures were reviewed and approved by the University College London Research Ethics Committee.

### Materials

#### Subjective measures

Binge eating symptoms were assessed via the BES^53^ (score ≥ 17 required for participation); and Eating Disorder Examination Questionnaire (EDE-Q^54,55^). The Yale Food Addiction Scale (Y-FAS) was used to assess addictive-like responses to food^56^. Susceptibility to food craving was assessed using the Power of Food Scale^57^ and general and state craving in response to HPFs by the food craving questionnaire-trait (FCQ-T) and state forms, respectively (FCQ-S)^58^. As depressed mood is comorbid with binge eating, we measured differences in recent depression with the Beck Depression Inventory (BDI^59^). Impulsivity; a putative predictor of binge behaviour, was indexed using the Barratt Impulsiveness Scale (BIS^60^).

A calendar-based self-report Timeline-Follow-Back measure was used to measure subjective binge frequency^61^, where participants reported the incidence of *subjective* binges; defined as ‘e*ating an unusually large amount of food with the subjective feeling of loss-of-control’*. This was confirmed by completion of a daily food diary via the *MyFitnessPal* app. Participants were asked to log everything they consumed for one week prior to session 1 (baseline), from session 2 to session 3 (post-intervention) and post-session-3 (follow-up). From this, total daily calories, carbohydrates, fats and sugars were calculated.

#### HPF Cue reactivity and ‘taste test’

The procedure is outlined in detail in the Supplementary Information. Briefly, ‘pleasantness’, ‘desire to eat’ and ‘likelihood of bingeing on’ was assessed for 18 HPF and 18 LPF images on a 0–100 scale. From this task, individualised HPF and LPF images (four of each) were selected per- participant, for later use in the visual probe and Go/No-Go tasks based on highest and lowest reward reactivity ratings. Prior to image rating, participants selected a preferred HPF snack food item from a ‘menu' and were told they would eat this after rating some food images, in a sham ‘taste test’. The selected food was placed in front of the participant and visible during the ratings of all food images and at the end the picture rating, was itself rated for ‘*desire to ea*t’ and predicted ‘e*njoyment*’ pre-consumption and its taste attributes, true ‘*enjoyment*’ and ‘*wanting more*’, post-consumption. The food was consumed according to on screen prompts requiring participants to ‘pick up food’, ‘prepare to eat’ and ‘eat the food’.

#### Go/No-Go Task

Response bias to binge foods was both assessed and retrained via a Go/No-Go task, adapted from Houben and Jansen^42^ and following previous research^38,62^. Full task details are given in the Supplementary Information and Ref.^63^. An ‘assessment version’ of the task was used in Sessions 1 and 3 and a ‘modification version’ on Session 2 (‘intervention' session). Task parameters were identical in both versions except HPF binge foods were paired with ‘No-go’ responses and LPF images paired with ‘Go’ responses on 100% trials in the ‘modification’ version. The ‘sham’ version of the Go/No-Go task on session 2 was simply the ‘assessment’ version; with parity between requirement for Go- or No-go responses for all stimulus types (HPF binge food, LPF or filler). Assessed indices of response bias were error rates, median reaction times, sensitivity (d-prime) and response bias (criterion *C*), indexing bias to ‘go’ to images regardless of response requirement^42^.

#### Visual probe

Eye-tracking in a dot-probe task was used to assess attentional bias to the self-selected LPF and HPF stimuli. All food images were paired with matched non-food images and dwell time and first fixation latency were calculated as indices of sustained and automatic attention, respectively. Details in Supplementary Information.

#### Binge memory retrieval and no-retrieval control

Participants in the BMR + RIT and BMR + sham groups underwent *Binge Memory Retrieval (BMR)* which followed a procedure parallel to those we have used successfully in previous studies on maladaptive reward memory reconsolidation^48,64^. The BMR procedure was introduced to the participants as a repeat of the session one ‘taste test’ (i.e. cue reactivity) task. Again, participants selected their favourite food from the ‘menu’ and were instructed that they would consume this after rating images. The presented images were the participant’s four highest-rated ‘binge cues’. They then rated their predicted enjoyment and ‘desire to eat’ their selected food. Following this, the on-screen consumption prompts read as before. The final prompt, however, read ‘*Stop, put food down’* at which point the food was taken away. Participants were thus prevented from consuming their anticipated food reward, putatively engendering a cognitive prediction error.

Participants in the NR condition followed the same procedure as BMR, except: (1) the binge food cues were replaced with the lowest-rated LPF food images from the cue reactivity task (2) Instead of selecting their favourite HPF from the menu, participants were given a non-binge LPF (celery sticks) and told they would eat this after rating food images. Thereafter, the image and food ratings and prompt screens were identical to the BMR procedure, including the prediction error procedure. The NR procedure was designed to match the BMR as closely as possible without (re)activating binge food reward memory.

### Procedure

After screening, participants attended three lab sessions and (remotely) provided follow-up data on four additional occasions (+ 2 week, 3 months, 6 months, 9 months). Prior to lab sessions they fasted from solid food (4 h) and abstained from caffeine (2 h). All lab sessions were conducted between 1 and 5 p.m. Written informed consent was given at the start of Session 1, following eligibility screening. The full procedure is outlined in detail in the Supplementary Information.

#### Session 1

Baseline demographic, questionnaire, biological (including blood glucose, blood pressure, weight & height for BMI calculation) and eating-related measures were obtained (see *supplement* for full list). In addition, state measures of food craving (FCQ) and hunger (hunger ruler) were assessed followed by the cue reactivity procedure and the assessment version of the Go/No-Go task. Finally, they completed the visual probe task.

#### Session 2 (session 1 + 48 h)

After repeating the biological and state measures from session 1, participants then completed the BMR or NR procedure as appropriate to their random group allocation. As with our previous studies^48,49^, following the BMR or NR procedure, participants completed high-load working memory tasks (prose recall from the Rivermead battery and digit span forwards and backwards), to ensure cognitive disengagement from the food cues. Following completion of these ‘distractor’ tasks (~ 5 min), participants began the ‘RIT’ or ‘sham’ version of the Go/No-Go task, followed by FCQ-state and ‘hunger ruler’.

#### Session 3 (session 2 + 7 days)

The *session 3* procedure was identical to *Session 1*, except the participants did not complete the BIS, BIS/BAS or BDI scale.

#### Follow-up

At 2 weeks, 3, 6 and 9 months following *Session 3*, participants remotely completed the BES, EDE-Q, Y-FAS, TLFB of binges, TFEQ, PFS and rated each image used in the initial cue reactivity assessment task on the same metrics as in-lab.

### Statistical approach

In-lab continuous measures (cue reactivity rating data, Go/No-Go reaction times, oculomotor attentional bias and state questionnaire measures) were assessed with 2 [*Session*:Session 1 (pre-manipulation) v. Session 3 (post-manipulation)) × 3 [BMR + RIT, BMR + sham, NR + RIT] × mixed ANOVA. Power calculation was based on this model (see Supplementary Information for full data handling protocols, sample size calculation data and randomisation). For analysis of cue reactivity and Go/No-Go RT data, a factor of Cue Type (HPF, LPF, non-food filler) was also modelled. For error rate and accuracy data in the Go/No-Go task, generalized estimating equations with a loglinear link function were used due to the approximate Poisson distribution of the count data. For long-term follow-up data, linear mixed models (LMMs; for continuous, normally distributed data) and generalized linear mixed models (GLMMs; binge count data) were used, incorporating effects of *Group*, *Time point* (baseline, post-manipulation, 2 weeks, 3 months, 6 months and 9 months) and their interaction. Signal detection metrics criterion *C* (i.e. ‘g bias’ and ${d}^{^{\prime}}$ were calculated for the Go/No-Go task) and analysed with LMMs and gamma GLMM (following inspection of data distribution). For tests of baseline trait, biometric and demographics variables, where group differences were not hypothesised, the false-discovery rate (FDR^65^) adjusted alpha level was applied. Post-hoc tests following omnibus tests were adjusted using the Sidak correction. Data were collected by LS and EC and analysed blind by RKD, using a code generated by SKK.

### Ethical approval

The authors assert that all procedures contributing to this work were approved by and comply with University College London Research Ethics Committee’s ethical standards on human experimentation and with the Helsinki Declaration of 1975, as revised in 2008*. *ISRCTN Registration Identifier: ISRCTN13262256. Open Science Framework Pre-registration: https://osf.io/82c4r/.

## Results

Descriptive statistics for key variables across groups are given in Table 1. Groups were very similar on assessed demographic variables, being typically in their early 20 s and in higher education. BES scores verified subjective binge-eating status and the PFS, TFEQ and FCQ indicated relatively high reactivity to food, emotional/uncontrolled eating and food craving indicating the sample displayed robust maladaptive reward responses to food. There was a trend for greater BMI in *BMR* + *RIT* than the other groups, due to three individuals with particularly high BMI (~ 37). There was also a trend for a difference (BMR + Sham > BMR + RIT) in the uncontrolled eating subscale of the TFEQ. Neither of these differences approached significance at FDR-corrected alpha. Groups were otherwise similar on baseline variables.

**Table 1:** Descriptive statistics of demographic variables at baseline. Values represent mean ± SD or counts. F-tests are one-way ANOVA with df 2.87.

Measure	Subscale	BMR + SHAM	BMR + RIT	NR + RIT	F	Benjamini–Hochberg Adjusted p value	Sig (uncorrected)
Sex	(N female/male)	23/7	22/8	22/8			
Age		21.24 ± 2.12	21.73 ± 1.8	21.02 ± 1.72	1.134	> 0.999	0.327
Education (years)		15.7 ± 1.92	16.13 ± 2.81	16.07 ± 2.672	0.263	0.998	0.77
STAI		47.18 ± 10.01	45.73 ± 10.63	45.3 ± 11.61	0.239	0.951	0.788
BDI		14 ± 7.7	13.27 ± 7.46	13.97 ± 8.48	0.081	> 0.999	0.922
BIS/BAS	BIS	13.32 ± 3.46	11.5 ± 3.14	12.4 ± 2.33	2.664	0.887	0.076
	BAS drive	7.93 ± 1.96	8.27 ± 2.57	8.57 ± 2.46	0.532	0.982	0.589
	Fun seeking	6.82 ± 2.29	7.3 ± 2.07	7.03 ± 1.54	0.424	0.957	0.656
	Reward responsiveness	7.68 ± 2.09	7.43 ± 2.05	7.3 ± 1.86	0.266	> 0.999	0.767
B.I.S		68.18 ± 7.76	64.63 ± 10.27	68.07 ± 10.11	1.338	> 0.999	0.268
BES		24.82 ± 6.32	24.67 ± 6.84	23.3 ± 7.33	0.441	0.982	0.645
EDE-Q		1.2 ± 0.58	1.33 ± 0.48	1.13 ± 0.61	1.008	> 0.999	0.369
Yale Food Addiction Scale	Clinically significant Impairment (N meeting)	2	2	4	0.537	> 0.999	0.586
	Symptom count	1.6 ± 1.57	1.47 ± 1.25	1.9 ± 1.63	0.665	0.952	0.517
Power of Food Scale	Food present	15.36 ± 3.29	15.03 ± 2.51	15.63 ± 3.6	0.27	> 0.999	0.764
	Food available	21.36 ± 5.21	21.03 ± 4.33	21.27 ± 5.46	0.032	0.968	0.968
	Food tasted	17.61 ± 4.4	17.97 ± 3.74	17.87 ± 4.91	0.052	0.978	0.95
TFEQ (R-18)	Uncontrolled eating	60.98 ± 9.9	54.07 ± 9.36	58.89 ± 13.28	3.028	0.945	0.054
	Cognitive restraint	59.92 ± 13.21	57.04 ± 19.46	62.04 ± 16.45	0.682	0.988	0.508
	Emotional eating	74.6 ± 22.19	74.81 ± 25.59	65.93 ± 22.59	1.378	> 0.999	0.258
Food craving questionnaire	Desire	10.71 ± 3.03	9.9 ± 3.29	11.2 ± 2.4	1.507	> 0.999	0.227
	Reinforcement	10.36 ± 3.07	9.77 ± 3.7	10.8 ± 2.7	0.794	0.995	0.455
	Relief	9.54 ± 3.12	9.43 ± 3.2	9.9 ± 2.02	0.225	0.932	0.799
	Control	9.43 ± 2.89	8.7 ± 3.01	10.13 ± 2.4	2.001	> 0.999	0.142
	Hunger	11.14 ± 2.77	11.47 ± 2.05	11.97 ± 1.94	0.974	> 0.999	0.382
	Total	51.18 ± 13.08	49.27 ± 13.15	54 ± 7.34	0.483	0.985	0.619
TLFB mean binges/day	(all previous 2 weeks)	0.44 ± 0.46	0.49 ± 0.37	0.46 ± 0.47	0.074	0.985	0.929
N binge days		4.73 ± 3.89	5.53 ± 3.37	4.37 ± 3.31	0.855	> 0.999	0.429
N binges total		7.1 ± 8.74	7.37 ± 5.76	6.3 ± 6.51	0.182	0.942	0.834
g Sugar/day		63.91 ± 24.6	86.04 ± 89.63	58.4 ± 32.37	1.751	> 0.999	0.18
g Carbs/day		204.39 ± 61.33	220.36 ± 72.42	191.58 ± 62.46	1.279	> 0.999	0.284
g Fat/day		40.9 ± 19.3	44.37 ± 16.89	47.19 ± 19.48	0.722	> 0.999	0.489
KCalories/Day		1926.68 ± 1019.29	1949.21 ± 637.24	1708.475 ± 482.6	0.852	> 0.999	0.43
BMI		23.03 ± 3.95	25.94 ± 5.36	23.36 ± 4.42	3.621	> 0.999	**0.031**
Fasting glucose		5.04 ± 0.55	5.13 ± 0.65	5.13 ± 0.55	0.24	0.984	0.787
Systolic blood pressure/mmHg		106.63 ± 15.18	105.4 ± 10.29	105.3 ± 11.07	0.799	> 0.999	0.453

Significant values are in bold.

### Short-term effects of RIT and BMR (in-lab measures)

Pre-manipulation, Go/No-Go task commission errors (False Alarms) were greater for both types of food stimuli (LPF and binge) than non-food stimuli. See Supplementary Information for full analyses. Error rates were examined across *Group*, *Session* (pre-manipulation vs post-manipulation), *Stimulus Types* (Binge, LPF, non-food filler) and *Error Type* (misses and false alarms). In line with analysis of baseline data, main effects of *Stimulus Type* (χ^2^(2) = 82.194, *p* < 0.001), *Error Type* (false alarms > misses): χ^2^(1) = 6.404, *p* = 0.011 and their interaction (χ^2^(2) = 13.013, *p* = 0.001) were found. The four-way interaction of Group, Stimulus Type, Error Type and Session was also significant. A three-way *Stimulus Type* × *Session × Error Typ*e interaction was present in all groups, although simple effects within each Group showed a change in response to Binge food stimuli only in *BMR* + *RIT* (see Table 2, top). At baseline, *BMR* + *RIT* showed significantly more false alarms than misses to binge food images (χ^2^(1) = 18.043, *p* < 0.001), however this was abolished post-training (χ^2^(1) = 1.222, *p* = 0.269).

**Table 2:** Simple effects analyses for interpretation of 2 way Stimulus × Error Type and Session × Stimulus Type interactions. P values are sequential Bonferroni-corrected.

Group	Stimulus type	Wald χ^2^ (3)	*P* value	Interpretation Session 1 vs 3
**Session × Error Type interaction: contrasts within each level of group and stimulus type**				
BMR + Sham	Binge	2.954	0.399	
	LPF	3.200	0.362	
	Filler	0.718	0.869	
BMR + RIT	Binge	27.707	**< 0.0005**	Reduction in false alarm rate
	LPF	4.840	0.184	
	Filler	11.541	**0.009**	Reduction in miss rate
NR + RIT	Binge	4.613	0.202	
	LPF	5.613	0.132	
	Filler	7.102	0.069	

Group	Error type	Wald χ^2^ (5)	*P* value	Interpretation session 1 vs 3
**Session × Stimulus Type interaction: contrasts within each level of group and error type**				
BMR + Sham	Miss	22.993	**< 0.0005**	Decreased binge food miss rate
	False alarm	13.948	**0.016**	Increased binge food false alarm rate
BMR + RIT	Miss	25.536	**< 0.0005**	Increased binge food miss rate
	False alarm	6.136	0.293	
NR + RIT	Miss	14.883	**0.011**	Decreased binge food and filler false alarms
	False alarm	8.058	0.153	

Significant values are in bold.

To qualify this effect, *Session × Stimulus Type* interactions were assessed within each Group and Error Type (see Table 2, bottom). This showed a significant increase in binge-food ‘misses’ from session 1 to session 3 in *BMR* + *RIT*, but a significant decrease in misses (i.e. greater response to binge food) in *BMR* + *Sham*, indicating potential worsening of approach bias in this group. In *NR* + *RIT*, there was a significant decrease in false alarms on binge food ‘no-go’ trials and a decrease in false alarms to filler images.

#### Signal detection measures

##### Criterion C

A 3 (Group) × 2 (Session: pre-manipulation, post-manipulation) × Stimulus Type (Binge, LPF, filler) factorial linear mixed model with bootstrapped parameter estimates found main effects of *Stimulus Type* [F_(2,450)_ = 3.59, *p* = 0.028] and a *Group × Session × Stimulus Type* interaction [F_(4,450)_ = 3.011, *p* = 0.018]. The 3-way interaction was investigated through examination of *Session × Group* interactions for each *Stimulus Type*. This revealed a Session*Group interaction for *binge images* only.

**Figure 1:**
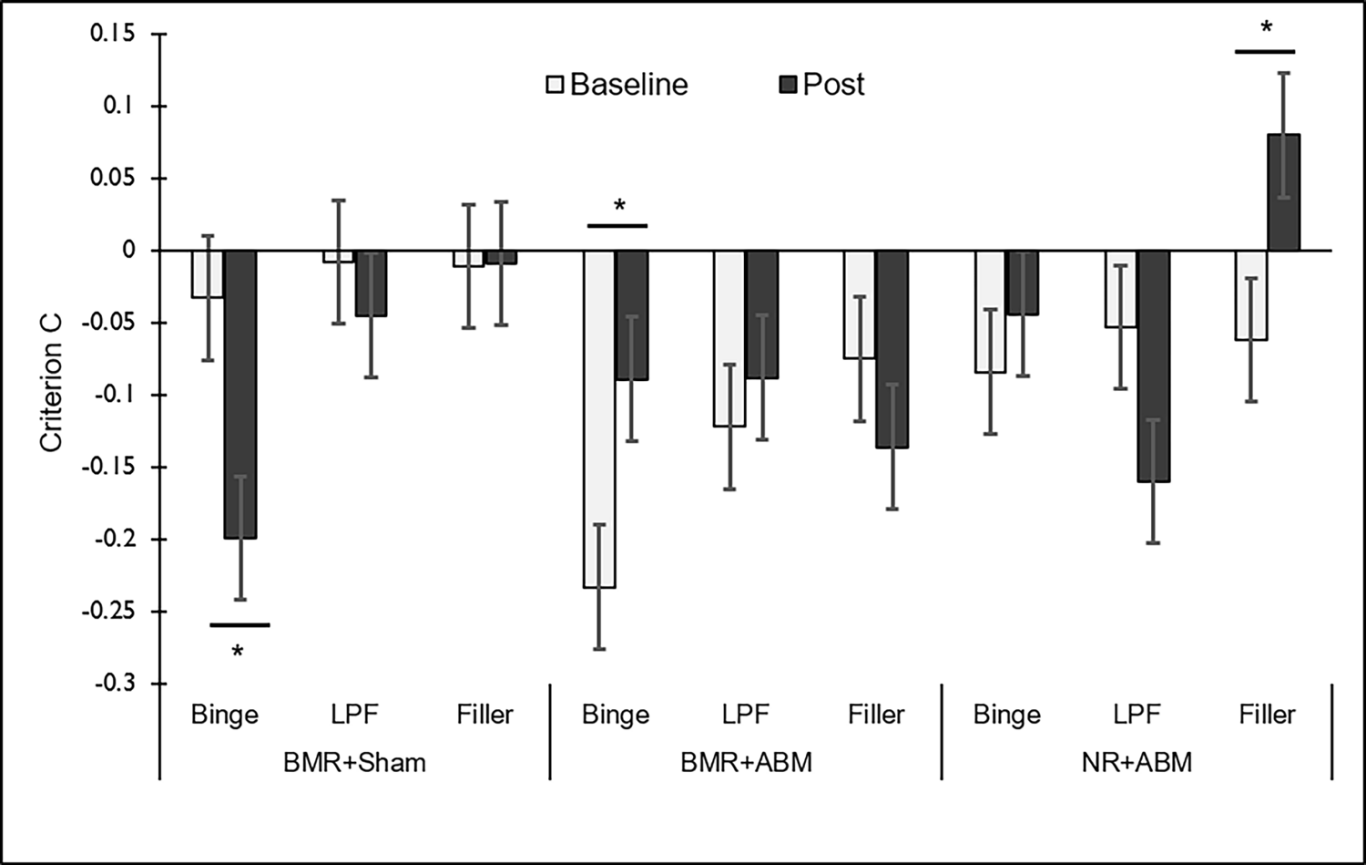
Changes in commission bias (Criterion C) across study sessions. Reductions in binge food commission (‘go’ bias) were seen in *BMR* + *RIT*, along with a shift in bias towards non-food images in *NR* + *RIT*. *BMR* + *Sham* showed an unfavourable shift in bias towards binge images, in line with a worsening of ‘go bias’ due to sham training. Bars are group mean ± SEM. *p < 0.05.

In *BMR* + *Sham*, there was a significant *worsening* of response bias to food, reflected in a reduction in *C* for binge images from session 1 to session 3 [F_(1,90)_ = 6.14,*p* = 0.015]. In *BMR* + *RIT*, there was a significant *reduction* in bias to binge images (increase in *C* towards 0) [F_(1,90)_ = 4.635, *p* = 0.034]. In *NR* + *RIT* there was no statistically significant change [F_(1,90)_ = 3.153,*p* = 0.079]. This is possibly evidence of a beneficial response in *BMR* + *RIT*, although it should be noted that this group showed the greatest bias to binge images on *Session 1,* indicating potential baseline dependency effects. This effect is shown in Fig. 1.

##### D prime (d′)

As with overall accuracy, *d*′ scores were highly skewed (z > 4 in most cases), indicating ceiling-level performance with regards to go/no-go signal sensitivity. For this reason, *d′* scores were analysed using a gamma generalized linear mixed model, including factors of *Group, Stimulus Type* and *Sessio*n factorially. This yielded a main effect of *Stimulus Type* only [F_(2,522)_ = 4.124, *p* = 0.016], indicating lower *d′* scores (reflecting greater false alarm rate) to binge food images vs. non-food filler images [*t*(522) = 2.783, *p* = 0.017], but no difference between HPF and LPF stimuli [*t*(522) = 0.766, *p* = 0.444].

##### Reaction time data

At baseline, median reaction times on (correct) ‘Go’ trials indicated an effect of *Stimulus Type* [F_(2,174_^)^ = 8.447, *p* < 0.001, *η*^*2*^_*p*_ = 0.089], that was invariant across groups [*Stimulus Type × Group* interaction: F_(4,174)_ = 1.948, *p* = 0.105, *η*^*2*^_*p*_ = 0.043]. Responses were faster to both types of food images (HPF and LPF) than non-food filler images [Helmert F_(1,87)_ = 14.82, *p* < 0.001, *η*^*2*^_*p*_ = 0.146], but not different between HPF and LPF images [Helmert F_(1,87)_ = 0.089, *p* = 0.766, *η*^*2*^_*p*_ = 0.001]. Thus there was an overall faster response to food images in the study sample, but not specifically to HPF ‘binge’ foods. A general speeding of responses between sessions 1 and 3 indicated practice effects [F_(1,87)_ = 32.643, *p* < 0.001, *η*^*2*^_*p*_ = 0.273], but there were no interactions nor group effects.

#### Oculomotor attentional bias (visual probe)

Dwell time assessment of HPF/binge food vs LPF food images found no evidence for differential sustained attention to binge-food images above any food image per se [main effect of image type F_(1,85)_ = 2.79, *p* = 0.099, *η*^*2*^_*p*_ = 0.032]. Equally, this did not vary pre-post manipulation [Session* × *Image Type F_(1,85)_ = 0.7, *p* = 0.792, *η*^*2*^_*p*=_0.001] or across Groups [*Session × Image Type × Group* F_(2,85)_ = 0.43, *p* = 0.65, *η*^*2*^_*p*_ = 0.01]. Dwell time on long-latency trials (2000 ms) incorporates early automatic and later conscious control of visual attention. Indeed, significantly reduced latencies to *first fixation* on binge food images was observed (a measure of *automatic* attentional capture) vs LPF images [main effect of image type [F_(1,85)_ = 27.508, *p* < 0.001,* η*^*2*^_*p*_ = 0.245. Combined with the lack of difference in dwell time, this suggested that following initial (automatic) attentional capture, participants deployed effortful visual avoidance strategies to disengage attention from binge food images.

#### Food craving questionnaire (state)

*Session* (1 vs 3)* × Time* (pre vs. post cue reactivity)* × Group* ANOVA yielded a reduction in general food craving from *Session 1* to *Session 3* [F_(1,82)_ = 4.058, *p* = 0.047, *η*^*2*^_*p*_ = 0.047], that did not significantly differ between groups. The desire subscale showed a *Session × Time × Group* interaction [F_(2,82)_ = 4.273, *p* = 0.017, *η*^*2*^_*p*_ = 0.094]. This was found to be driven by a decrease in food desire across sessions in *BMR* + *Sham* at the post taste-test time point [F_(1,82)_ = 7.173, *p* = 0.007, *η*^*2*^_*p*_ = 0.086]. Conversely, a decrease in pre taste-test desire across sessions was seen in *NR* + *RIT* [F_(1,82)_ = 6.498, *p* = 0.013, *η*^*2*^_*p*_ = 0.073]. There was a reduction in the control subscale from session 1 to 3 [F_(1,82)_ = 4.372, *p* = 0.04, *η*^*2*^_*p*_ = 0.051], that did not differ between groups. The same was found for the hunger subscale [F_(1,82)_ = 8.067, *p* = 0.006, *η*^*2*^_*p*_ = 0.09]. No significant change was observed on the relief or reinforcement subscales.

#### Cue reactivity

Ratings of ‘likelihood to binge on’ depicted food images (HPF vs LPF/Session 1 vs. Session 3) showed a main effect of image type (HPF > LPF: F_(1,85)_ = 564.630, *p* < 0.0005, *η*^*2*^_*p*_ = 0.869), validating the use of HPF and LPF images as representing binge foods and non-binge foods, respectively). A modest *Group × Session* interaction was observed [F_(2,85)_ = 3.931, *p* = 0.023, *η*^*2*^_*p*_ = 0.085]. While there were no group differences on *Session 1*, on *Session 3* (post-manipulation), both *BMR* + *RIT* [*t*(57) = 2.996, *p* = 0.011, *d* = 0.78] and *NR* + *RIT* [t(59) = 2.74, *p* = 0.022, *d* = 0.71] reported lower likelihood of bingeing on any depicted food than *BMR* + *Sham*. A marginal *Session × Group* interaction was also observed for food images’ impact on *urge to eat* ratings [F_(2,85)_ = 3.167, *p* = 0.047, *η*^*2*^_*p*_ = 0.069]. *BMR* + *RIT* reported lower ‘urge to eat’ than *BMR* + *Sham* on both sessions, however *BMR* + *Sham* showed lower urge to eat than *NR* + *RIT* on session 3 [*t*(59) = 2.89, *p* = 0.015, *d* = 0.75]. This was largely due to an *increase* in desire to eat ratings in *NR* + *RIT* from session 1 to session 3 [F_(1,85)_ = 4.037, *p* = 0.48, η^2^_p_ = 0.045].

#### Long-term disorder-relevant outcomes

##### Binge Eating Scale

The mixed modelling approach for the BES was supported by significant variance in intercepts (*σ*^*2*^ = 37.315, Z = 5.519, p < 0.001). A significant effect of *Time* (baseline (session 1), post-manipulation (session 3), 2 weeks, 3 months, 6 months, 9 months) was observed, indicating reduction in symptom severity across the study, but no effects of Group nor interaction (see Table 3). Despite significant variance in the *Time* effect: (*σ*^2^ = 8.178, SE = 3.617, Z = 2.261, *p* = 0.024), modelling *Time* as a random effect worsened BIC- assessed model-fit (3069.464 → 3072.779) and did not alter interpretation of any model terms. Bayes Factors (see supplement for calculation) provided substantial evidence in favour of the null hypothesis of no *Group × Time* effect (BF_01_ = 83). Bayes Factors calculated at follow-up time points for ANOVA across groups and for Welch’s *t-*tests contrasting RIT vs. sham (with the alternative hypothesis of *lower* BES scores in the RIT conditions), similarly provided evidence in favour of no differences post-intervention, but the latter were uninformative at subsequent time-points owing to reduced sample size. BF_01_s for these contrasts are given in Table 3*.*

**Table 3:** Linear mixed model parameters for Binge Eating Scale scores and Bayesian ANOVA and Welch’s t-tests for group differences and the contrast RIT > sham at all post-intervention time points. *BIC* Bayesian Information Criterion, *− 2LL* = − 2 log likelihood.

Model fit	− 2LL = 2910.393	BIC = 3069.464		
Fixed effects	df	F	Sig	
Intercept	1, 95.531	700.895	< 0.0005	
Group	2, 95.469	0.450	0.639	
Time	5, 148.131	24.131	< 0.0005	
Group × time	10, 148.267	0.576	0.832	

Time point	One-way ANOVA BF_01_	Error %	RIT vs. Sham BF_01_	error %
Post-intervention	9.35	0.038	4.35	~ 0.010
2 week	6.94	0.027	2.1	~ 0.008
3 month	2.75	0.035	1.01	~ 0.005
6 month	7.75	0.026	2.97	~ 0.014
9 month	5.85	0.029	1.95	~ 0.011

##### ED symptomatology, craving and food addiction

*FCQ* total scores also reduced across *Time* from baseline to all subsequent time points [F_(5,107.26)_ = 22.719, *p* < 0.001]. However, the *Group × Time* interaction was non-significant [F_(10,107.351)_ = 0.759, *p* = 0.667]. Despite significant variance in the *Time* slopes (z = 3.178, *p* = 0.001), treating *Time* as a random effect worsened overall model fit. Similarly, negative binomial GLMM on YFAS symptom count scores showed an effect of *Time* [F_(4,133)_ = 2.468, *p* = 0.048], indicating a reduction in food addiction-like symptoms from baseline to all follow-up time points (all *ts* ≥ 2.066, *ps* ≤ *0.0*39). The *uncontrolled eating* subscale of the TFEQ paralleled these effects, with significant reductions across time from Baseline to all subsequent time points [F_(5,104.368)_ = 7.663, *p* < 0.001]. However, no significant *Group × Time* interaction was observed [F_(10,104.382)_ = 1.2, *p* = 0.299]. Measures of disordered eating symptomatology were thus highly consistent in their pattern and supported a lack of *Group* effects.

##### Binge frequency

Poisson GLMM found a significant main effect of *Time* was found [F_(5,84)_ = 16.149, *p* < 0.001]. This represented a reduction in binge episodes from baseline to all subsequent time points in all groups. No significant effects of *Group* [F_(2,94)_ = 0.028, *p* = 0.972] or *Group × Time* interaction were found [F_(10,85)_ = 0.862, *p* = 0.572]. The same pattern of results was found when modelling mean daily binge calories (using a gamma GLMM with log link). The intervention thus had no differential impact on bingeing behaviour (Table 4). Bayes factors favoured the null in one-way ANOVA at each time point and favoured no difference, or were inconclusive, for t-tests between RIT and sham (see Table 4).

**Table 4:** Changes in mean binge episode frequency from baseline to all post-intervention time points. T-tests are vs. baseline. Bayes Factors are for between-groups comparisons at post-intervention time points (ANOVA or Mann–Whitney U). Null hypothesis = (no group difference) vs alternative (group means unequal for ANOVA; RIT bingeing < Sham bingeing in Mann–Whitney). Bayes factors > 3 are considered evidence in favour of the null.

Time point	D baseline	t	df	p value	95% confidence interval	
Post-interventon	− 2.407	− 5.877	104	< 0.0001	− 3.219	− 1.595
2 weeks	− 2.573	− 5.324	121	< 0.0001	− 3.529	− 1.616
3 months	− 3.133	− 6.377	133	< 0.0001	− 4.104	− 2.161
6 months	− 2.867	− 4.918	115	< 0.0001	− 4.022	− 1.713
9 months	− 2.969	− 5.616	131	< 0.0001	− 4.015	− 1.923

## Discussion

Learned cognitive biases have been posited to be an important factor in maintaining binge eating behaviour^66^ and a prime target for therapeutic intervention. This study sought to examine the possible augmentation of therapeutic efficacy of food response inhibition training (RIT) via putative ‘reconsolidation-update’ mechanisms in sub-clinical binge eating young adults. Participants generally showed robust reductions across the spectrum of maladaptive binge behaviours assessed. However, we found very little evidence for beneficial effects of RIT on either short-term indices of response biases (Go/No-Go and visual probe and cue reactivity), nor any clinically-relevant measures of eating disorder symptomatology (binge episodes, BES, YFAS). Equally, a retrieval procedure designed to elicit memory destabilisation prior to bias retraining produced minimal augmentation of subsequent RIT effects.

Despite evident bingeing behaviour and cognitive symptomatology, our sample displayed extremely high performance accuracy on the Go/No-Go task, indicating relatively little in the way of premorbid response inhibition deficits to binge cues and possibly restricting the potential impact RIT *a* *priori* . Via signal detection analysis, we observed significant, albeit modest, ‘go’ biases to *all* food stimuli (both HPF/binge foods *and* LPF/non-binge foods) and automatic visual attentional capture by HPFs in eye-tracking metrics. We found greater reductions in response bias on the Go/No-Go task in *BMR* + *CBM*, but insufficient evidence that these differences were substantively related to eating behaviour.

While promising short-to-medium-term effects of food inhibitory control training have previously been seen in laboratory studies in ‘healthy volunteers’^43,67,68^ and ‘obese’ individuals (primary researchers’ own terms)^69^, both this and recent research has observed modest effects in the majority of tested longer-term clinical endpoints and eating behaviour in disordered eating groups^46,70–73^. These inconsistencies may be due to different effects across (dis)ordered eating populations, focus on short-term (lab-based) vs. lasting effects and training parameters and control procedures, which have been shown to be key determinates of food response inhibition train effects^45^.

We adapted the Go/No-Go task which effectively reduced chocolate ‘go’ bias and consumption in previous research^42^. It is possible that the greater diversity of high-palatability food (HPF) stimuli used here were less evocative of response biases than chocolate-only stimuli, producing more heterogeneous responses. However, the HPF stimuli in our study were individualised based on idiosyncratic ratings of the most rewarding images from a pool of food images with high normative ratings for reward value^74^. Reactivity to these images should therefore be as high as could be expected within the bounds of an experimental setting. Eye-tracking data confirmed that HPF stimuli were salient, robustly inducing automatic *visual* orienting^75^ to a greater extent than low palatability foods, an index predictive of actual food intake^76^.

A more compelling explanation for the disparate findings is the inflation of previous studies’ effects by suboptimal choice of (or lack of) control for inhibitory training procedures^34^. Earlier studies on RIT in chocolate consumption employed a ‘control’ condition that pairs chocolate images with ‘go’ responses. This ‘go control’ is not inert, in that it may *increase* approach bias to chocolate images and maximise the apparent effect of RIT by artificially inflating the difference between conditions. Indeed, studies using such ‘opposing control’ conditions tend to show significant effects, whereas those using true ‘sham’ training, (50/50 Go/No-Go) do not^78,79^ but  see Ref, an effect verified experimentally by Adams *et al*.^45^. As ‘food → go’ training may worsen overeating symptoms, it is not a clinically viable option as a control condition. For further rationale on for the sham CBM used here, see the Supplementary Information. Despite this, some studies have found positive effects with appropriate ‘sham’ controls.

Most studies on RIT employ immediate or 24-h post tests and it is possible that the 1-week delay between training and test we used prevented us from observing any immediate effects of training. However, we have observed effects in harmful drinkers from a similarly brief, single post-retrieval interventions that have been evident at 1-week and at least 9-months afterwards. If RIT effects are only observable in laboratory measures and at short latencies, we must question the comparative clinical utility of such an approach.

Clearly, null findings with a specific form of CBM (RIT) here do not preclude possible effects of other CBM modalities, such as approach bias modification, which, at least within the domain of AUD, have shown more consistent clinical effects. It is possible that alternative forms of CBM would have been more effective than the RIT used in the current study. The relative efficacy of these different modalities in changing maladaptive eating behaviour remains an open question in need of assessment. However, an examination of experimental evidence published since pre-registering this study and collecting the data (https://osf.io/hjtw3) indicates that this pattern of inconsistent findings is not specific to RIT, but is reflected in the broader literature examining CBM in binge and over-eating^46,71,80,81^, calling us to question the key moderators and potential therapeutic impact of cognitive bias modification. More robust effects of CBM generally, have been found for alcohol use disorders^82^, suggesting effects may be reward-domain specific. Indeed, although the authors of a recent narrative review concluded favourably for CBM across reward domains^78^, evidence for its efficacy in modifying eating behaviour has been questioned by authors of primary studies^79^ who note inconsistent findings and inappropriate CBM control groups. As a whole, therefore, the field would benefit from more consistent and well-controlled task design, larger randomised controlled studies with longer-term follow-up and direct assessment of the relative efficacy of different CBM modalities across different reward domains.

### Limitations

The aim of this study was to assess whether RIT efficacy could be catalysed by conducting retraining following ‘reactivation’ of maladaptive food reward memory, as we have shown for behavioural and drug interventions^48,50^. We did not find evidence of such effects, aside from in short-lived Go/No-Go task performance interpreting this as a reconsolidation-update effect would be tenuous. However, demonstrating therapeutic enhancement via maladaptive memory reminders is dependent upon a memory-targeting intervention having a minimum of standalone efficacy. Since CBM was largely ineffective, even in the short term (in-lab) measures of responding collected here, we are unable to make any conclusions as to whether food reward memories were successfully destabilised our retrieval procedure nor whether this could confer additive benefit in longer-term clinical outcomes to a standalone behavioural therapy for binge eating. Multiple sessions of training could be used, although one of the great appeals of a putative reconsolidation-based therapy is its single-shot nature.

Binge-eating individuals frequently *already* engage in compensatory strategies to regulate their weight and minimise binge episodes, including effortful inhibition of food approach, and avoidance of ‘trigger’ foods. Our eye tracking data support this notion. The complex relationship that binge eating individuals have with binge foods thus entails reward and approach, but also avoidance, self-criticism and shame^84^ following bingeing. If binge-eating individuals are already well-practiced in trigger food avoidance strategies, the potential for added efficacy of brief avoidance training may have been limited a priori. Identifying target sub-groups with high levels of baseline response bias may yield greater effects of retraining. While we have found minimal evidence in the current study to recommend RIT as a clinical intervention in binge eating, given the relative ease of its implementation (e.g. via smartphone apps), limited potential for harm when constructed correctly and potential to orient more attention to one’s eating behaviours and related cognitions, there may be a rationale to recommend pursuing RIT approaches in these groups.

Our study sample were not receiving treatment and binge eating behaviour was primarily assessed via self-report instruments, which may be considered sub-optimal. However, it was not our intention to diagnose binge eating in this study and we focussed upon adolescent sub-clinical binge eaters as a group in whom preventative, low intensity interventions might be usefully employed. The existence of the relevant disordered eating behaviours was further triangulated against other disordered eating measures and food logs. Regarding these, one reviewer noted that *MyFitnessPal* usage is prevalent among disordered eating populations^85^ and on pro-eating disorder forums, questioning the ethics of it use in the current setting. While there is no current evidence for a causal link between MyFitnessPal usage and eating disorders and the reductions in eating disorder symptomatology across all groups in the current study suggest it was not a cause of harm, future studies may instead wish to use recovery-focussed apps, such as Recovery Record.

### Strengths

We employed a highly rigorous randomised, pre-registered design including more appropriate control procedures than some previous studies, and comprehensive assessment of both short-term target cognitive processes and long-term eating behaviour and disorder symptomatology, with a follow- up period considerably longer than prior research. This allows us to fairly comprehensively reject the possibility of lasting intervention efficacy over a clinically-relevant timeframe. Doing so, we found no evidence for a lasting beneficial effect of inhibitory control training, either alone or when combined with pre-training maladaptive memory retrieval.

## Supplementary Information

Supplementary Information.

## Data Availability

Study data are available upon request from Ravi Das.
