# Supplementary material for: The effects of response inhibition training following binge memory retrieval in young adults binge eaters: a randomised-controlled experimental study
Source: Sci Rep. 2022 Jun 3;12:9281. doi: 10.1038/s41598-022-12173-w (PMC9166753; doi:10.1038/s41598-022-12173-w)
Supplement: Supplementary file 1 — Supplementary Information. [file 41598_2022_12173_MOESM1_ESM.docx]

**Supplementary Materials**

**Procedures:**

**Recruitment and Screening:** Participants were recruited via online advertisements and via specialised recruitment platforms (Call for Participants, SONA systems). No participants were recruited directly from NHS or private healthcare services. Following response to study advertisements, participants were contacted by telephone to be screened for eligibility.

**Inclusion criteria:** Ages 18-25, sub-clinical binge-eating defined as bingeing >1/month, Binge Eating Scale (BES) score >17, English speaking, healthy and neurologically normal.

**Exclusion Criteria:** Existing Clinician-diagnosed Eating Disorders, or any mental health disorder, use of psychiatric medication, BMI<18, compensatory purging behaviour, restricted diet (e.g. veganism, ketogenic diet or Jainism), diabetes, lactose intolerance.

Where scores on the BES or EDE-Q indicated likely clinical levels of eating disorders, respondents did not continue to participate, but were referred to NHS guidance on seeking treatment for eating disorders and advised to contact their GP for further advice.

Following eligibility screening and completion of written informed online consent, participants were allocated a participant number and given, individual access links to MyFitnessPal accounts, along with instructions for filling these diaries out for the week prior to their first study session. Following study completion (sessions 1 to 3), participants were reimbursed at a rate of £10 per hour, with an extra £5 incentive for each follow-up time point completed. All participant data were pseudonymised and stored in compliance with UCL’s data protection regulations and GDPR (2018). Approval for all study procedures was provided by the UCL Research Ethics Committee and all procedures were in line with the Declaration of Helsinki (2016).

**Randomisation and blinding:**

Randomisation was unblocked, using a random non-repeating sequence generated via random.org. Random numbers were assigned to participant numbers (1-90) in the order generated, then sorted in ascending order on the random number. Participant numbers were assigned numeric grouping codes (lowest 30 = 1, middle 30 = 2, highest 30 = 3), with codes randomly allocated to groups by SK (1 = BMR+Sham, 2 = BMR+RIT, 3, =NR+RIT). The randomisation code was blinded to the analyst (RK) until after completion of data analysis. Data collectors (LS, EC, GP) were blinded to RIT/sham condition, but could not be blinded to binge memory reactivation/no reactivation.

**Cue reactivity:**

Image Set preparation

A stimulus set of 36 food images (18 HPF and 18 LPF) was developed from the FoodPics database ^1^. In a baseline food cue ‘reactivity’ task, participants rated the ‘pleasantness’ of each image, their ‘desire to eat’ the depicted item and their likelihood of bingeing on the depicted item on a 100 point VAS. Four ‘binge food’ images and four ‘LPF’ images for each participant were selected on the basis of highest and lowest binge reward ratings for use in the visual probe and Go/No-Go tasks.

Fifty images from the Food-Pics database ^1^ were selected based upon the normative sample ratings for food reward (designated HPFs) and lowest reward norms ratings (designated LPFs). These were sent to a local pool of raters (N = 20) for piloting as to their pleasantness and reward value (Food-Pics was developed and validated in Austrian and German sample, where social norms regarding ‘high reward’ food have idiosyncratic differences to those in the UK). The 18 images scoring highest (HPFs) and lowest (LPFs) for food reward when combining normative ratings and ‘local’ pilot ratings were selected as the final stimulus set, along with eight non-food ‘filler images’ selected from FoodPics ^1^ on the basis of neutral normative ratings. For rating of images during the ‘cue reactivity’ task, all images were presented centrally on-screen in a fully randomised order in high resolution (800x800 pixels).

Participants rated each image on a 0-100 sliding scale for ‘How much I like’, ‘Current urge to eat’ and ‘Likelihood to binge on’ the depicted foods. All responses were made using an on-screen visual analogue scale from 0 to 100 with anchors ‘extremely unpleasant’ – ‘extremely pleasant’, ‘would hate to eat’ – ‘would love to eat’, ‘very unlikely’ – ‘very likely’ to binge, respectively. From these ratings, an overall ‘reward value’ score was calculated for each image with the following formula:

$$Reward value=pleasantness+urge+2(binge likelihood)$$

The value was weighted by the binge likelihood score on the basis that true ‘binge foods’ were the most appropriate to reactivate binge memory (BMR). Using the reward value ratings, the four highest and lowest scoring images were selected as individualised HPF and LPF stimuli, respectively, for use in the Go/No-Go and visual probe task.

**High palatability food ‘taste test’ menu:**

A selection of foods for the cue reactivity, binge memory reactivation and taste-test procedures were selected from popular and readily available high-palatability snack foods in the UK. These were selected to represent a balance of both sweet and savoury foods, have pre-portioned/pre-wrapped formats and be approximately equated in mass and calories. Nutritional information for these foods is given in table S1, below.

For the ‘taste test’ component of the cue reactivity task, participants were given their selected food prior to rating HPF/LPF image cues, then rated their ‘desire to eat’ and ‘anticipated enjoyment’ **of their selected food,** on a 100-point scale. They then ate the food following on-screen prompts reading ‘pick up the food’, ‘prepare to eat’ and ‘eat your food now’. On the final prompt, the participants were given as long as they wished to consume their food of choice while rating its taste attributes (‘sweetness’, ‘saltiness’, ‘crunch’, ‘greasiness’, ‘sourness’, and ‘bitterness’) on a set of VASs. The taste attributes were not of interest to the study outcomes and not analysed. Finally, after finishing the food, participants rated how much they enjoyed the food (‘pleasantness’ rating) and their ‘desire to eat more’ of it on a 0-100 scale. If any food was left, this was removed and later covertly weighed by the experimenter.

Table S1: Selection and nutritional information for foods included in the cue reactivity ‘taste test’ menu.

| **Food item** | **Manufacturer** | **Weight (g)** | **Calories** | **Fat (g)** | **Sugar (g)** | **Salt (mg)** |
| --- | --- | --- | --- | --- | --- | --- |
| Ready salted crisps | Walkers (UK) | 25 | 171 | 10.4 | 0.1 | 460 |
| Mini Babybel Original cheese | Bel Brands (UK) | 21 | 70 | 6 | 0 | 160 |
| Peperami mini original | Jack Link’s (Germany) | 10 | 40 | 1 | <0.5 | 410 |
| Angel slice cake | Mr Kipling (UK) | 33 | 139 | 6.1 | 13.1 | 190 |
| Mini Rolls (milk chocolate) | Cadbury Cakes (UK) | 27 | 115 | 6.2 | 11.4 | 180 |
| Maoam Stripes Minis | Haribo | 25 | 95 | 1.6 | 15 | 20 |
| KitKat Original 2 -finger | Nestlé (UK) | 21 | 104 | 5.1 | 10.6 | 50 |
| Lindor Milk Bar | Lindt & Sprüngli (UK) | 38 | 230 | 16.7 | 17.1 | 53.2 |
| Ritz Breaks Original | Mondelez UK | 31.6 | 146 | 5.7 | 2.9 | 400 |

**Go/No-Go Task details:**

This task was adapted from Houben & Jansen ^2,3^. Throughout the task, 500x500 pixel target stimuli (images) were presented centrally on-screen. On each trial, participants were required to either make a speeded key-press response (‘Go trials’) or to withhold this response (‘No-Go Trials’). ‘Go’ and ‘No-Go’ trial were indicated by a letter ‘f’ or ‘p’ overlaid on the trial image, with letter/response contingencies counterbalanced across participants. Responses were speeded via a short response window (1000ms). Three ‘Stimulus Types’ were used in the task: images of ‘HPF binge foods’ (k = 4, individualised on the basis of those rated highest in the Session 1 cue reactivity task); images of low-calorie, low palatability foods (k= 4 LPFs e.g. celery, lettuce, green peppers), or non-food ‘filler’ objects (k = 8). All images were taken from the FoodPics database (image codes available upon request from the authors) ^1^

Two versions of the Go/No-Go task were created, an ‘assessment’ version and a ‘modification’ version, that differed only in the contingency between Stimulus Types and response requirements. In the ‘assessment’ version of the task (used to assess bias on sessions 1 and 3), each Stimulus Type required an equal number of ‘go’ and ‘no-go’ responses (10 go & 10 no-go per image). Each image was thus presented 20 times, in a pseudorandomised order (random conditional on no more than two consecutive presentations of an image), yielding 320 total trials.

In the ‘modification’ version of the Go/No-Go task (‘RIT’, session 2), stimulus presentation parameters were identical to the ‘assessment’ version, except HPF binge foods were paired with ‘No-go’ responses and LPF images paired with ‘Go’ responses on 100% trials, to putatively train inhibitory ‘no-go’ responses to binge food images ^3^. Filler images were paired with ‘Go’ and ‘No-Go’ trials on a 50/50% schedule.

The ‘sham’/control version of the task (session 2) was simply the ‘assessment’ version of the Go/No-Go task, where there is parity between requirement for go or no-go responses for all stimulus types. The overall response requirements (N ‘go’ and ‘no-go’ trials) were identical in the ‘RIT’ and ‘sham’ tasks. Any differences represent an effect of the specific association of binge food images with inhibition, rather than just practice in inhibiting responses overall (see discussion, below for thy this is critical). Approach biases to the different stimulus types were indexed via error rate, median reaction time and criterion bias C; indexing bias to ‘go’ to images despite response requirement. ^3^

**Food Diaries**

Owing to the inaccuracy of retrospective food frequency questionnaires, daily food diaries were kept by creating individual accounts on the MyFitnessPal platform for the specific purpose of the study. MyFitnessPal consists of a user-curated foods database with macronutrient and caloric information per food gram, which calculates total daily intake from logged items. Participants were asked to log all foods consumed each day as they were consumed, or otherwise at the end of the day after the final meal or snack of the day. Participants were instructed on logging into the app and given advice on logging foods (i.e. enter raw weights and specific brand names, rather than generic).

Participants received the following instructions: on MyFitnessPal via email:

“Use this to **log your entire food intake and any binges that occur**(using the notes section)**for 7 days prior to your first lab session**. You will use this account to log all food intake for the full duration of the testing sessions (i.e. from 7 days before, and until 7 days after your final lab session).

Please also record binges in the notes section of this app by writing binges=#. Please do this even on days when binges=0.

Using the definition of a binge as to consume an unusually large amount of food in a sitting and experience loss of control over food intake."

Food diaries were monitored by the experimenters on a daily bases and reminders sent to participants on each day when no food was logged on the app. Caloric totals were calculated automatically by MyFitnessPal on the basis of 1g protein or carbohydrate = 4kcals and 1g fat = 9kCals. On study sessions 1 and 3, the experimenter cross-checked any blank entries on food diaries while the participants were in the study centre, in an attempt to improve completeness of reporting. Where blank entries were noted, participants were asked to retroactively complete these.

**Order of assessments (Sessions 1 & 3**):

Following witnessed, written informed consent (session 1 only), participants’ height and weight were taken using digital scales and a finger-prick blood glucose and blood pressure assessment was performed. They then completed basic demographic information and with the participants before completing the baseline questionnaire battery (TLFB, Y-FAS, BIS/BAS, FCQ (Trait), PFS, STAI-T, TFEQ-18, BDI, EDE-Q, BIS, FCQ (state), BES). Finally, participants completed the Go/No-Go task (assessment version) and visual probe task. Immediately prior and following the Go/No-Go task, participants were asked to rate their hunger on a ‘hunger ruler’; a 10-point visual scale with anchors ‘not at all hungry’ to ‘extremely hungry’. All questionnaire measures were completed electronically via Qualtrics software to minimise errors in digitising responses.

**Data Handling:**

Sample size: The study sample of N = 30/group was determined on the basis of high power (1-β = 0.95) to detect an interaction term in primary 2 x 3 mixed ANOVA with a moderate effect of η^2^_p_ = 0.06 and modest within-subjects correlation ρ = 0.5. This sample size would allow us to observe effects as small as η^2^_p_ = 0.037 at 1-β = 0.95 and effects as small as η^2^_p_ = 0.027 at 1-β = 0.8. We considered effects smaller than this to not be clinically meaningful.

Calculation of Signal Detection Indices

‘**Criterion’ C:** is an index of commission bias, calculated as – (Z Hits – Z false alarms)/2. In this case it represents the bias to ‘go’ to stimuli, whether appropriate to response requirements or not and therefore is hypothesised to provide a key index of approach biases to food images in the current sample. C is negatively tuned, with more negative scores indicating a commission (approach bias) and scores of 0 indicating no bias. This measure of response yielded far more normally distributed data than raw counts and was therefore appropriate for analysis with linear mixed modelling.

**D’** (‘d-prime’) is calculated as the z-normalised probability of **hits (z Hits)** – the z-normalized probability of false alarms **(z FAs)**. This gives an overall measure of signal detection that is independent of ‘go’ response biases (criterion). As such, it is a good measure of task-specific performance, as it indexes sensitivity and appropriate response to ‘go’ and ‘no-go’ instructions.

**Outliers:**

Where highly significant outliers were identified, these remained in the analysis subject to Winsorizing, where their values were replaced by the next highest (non-outlying) score + 1 unit. One high outlier in body mass index (BMI; BMR+RIT) was Winsorized. One highly outlying positive data point in the count of binge episodes (NR+RIT, time point 2) who reported 66 binges in a 7-day period was winsorized. Following winsorization, data were still over dispersed so a negative binomial mixed model was used.

**Bayes Factor calculation:**

To improve directional inference regarding null effects, where null effects were observed on key, disorder-relevant outcomes, Bayes Factors (BF_01_) were calculated. These represented the probability of the null over the alternative hypothesis) for one-way ANOVA on the post-intervention group differences in relevant score, or for t-tests or Mann-Whitney U tests comparing the effects of RIT (regardless of retrieval; i.e. NR+RIT and BMR+ RIT combined) vs. sham training. These were calculated using the BayesFactor package in R ^4^, which implements the Rouder et al method ^5^ with default JZS (Jeffrey) priors.

**RESULTS:**

Presence of food approach bias pre-intervention:

General Estimating Equations (GEEs) were used to assess counts of incorrect responses (i.e misses and false alarms) in the assessment version of the Go/No-Go task at baseline, as the data were counts and approximately Poisson-distributed. Incorrect responses were investigated as we hypothesised that errors in cognitive control better represented putative underlying biased processing than correct performance in line with task demands. GEEs were used due to favourable computational demands and performance in estimating fixed effects vs GLMMs ^6^. Model independent variables were Group, Trial Type (Go vs. No Go) and Stimulus Type (Binge food, LPF, non-food filler)

This analysis on Day 1 data yielded a Trial Type (No Go > Go) main effect: χ^2^(1)=7.892, p=.005, Stimulus Type main effect χ^2^(2)=50.647, p< .001 under a Stimulus Type x Trial Type interaction (χ^2^(2)=12.41, p=.002 ) and a Group x Trial Type interaction (χ^2^(2)=7.041, p= .03). Overall, more errors were made in response to non-food filler images (mean = 1.14(.138) CI _95%_ 0.9 to 1.44) than both binge food images (mean = .61(.113); CI_95%_ 0.42 - 0.87, p < .001) and LPF images (mean = .53(.076); CI_95%_  0.4 -0.7, p< .001). More errors were made on ‘no go’ trials (i.e. false alarms; mean = .91(.113); CI_95%_ 0.68 – 1.23) than ‘go trials’ (i.e. misses, mean = .56(.093); CI_95%_ 0.4 - 0.77). The Group x Trial Type interaction indicated that BMR+RIT made more errors on ‘No-Go’ trials (mean = .93(.142); CI_95%_  0.68 -1.25) than ‘Go trials’ (mean = . 31(.079); CI_95%_  0.19 -0.51, p = .001). Go and No-Go error rates did not differ in the other two groups. The Trial Type x Stimulus Type interaction indicated that there were more errors on No-Go trials (i.e. false alarms; = 1.016(.212); CI_95%_  0.67 -1.52) than Go trials (i.e misses= 0.36(.084); CI_95%_  0.23 -0.57)) for Binge images (p=.008). There was no difference in false alarm and miss rate for LPF (p > 0.999) and filler (p =.392) images. Compared to non-food filler images, misses were less frequent for both binge images (mean diff = -.99(.206), CI_95%_  -1.6 -0.39, p < .001) and LPF images (mean diff = -.86(.224), CI_95%_  -1.5 -0.21, p = .002)

However, in general performance on the Go/No-Go task was extremely high, with >96% accuracy in most cases. This ceiling effect questions the a priori assumption of robust of approach bias in the sample.

**Manipulation Checks:**

Binge Memory Reactivation

‘Surprise’ ratings following withholding/non-withholding of foods after the session2 BMR/NR procedure differed significantly between groups (Kruskall Wallis H =23.521, p<.001). The withholding of food in the two ‘prediction error’ groups successfully induced expectancy violation at the group level. There was, however, considerable variability in the amount of surprise induced in NR+RIT (see dotplot, below), as evidenced by a significant Levene’s test [F(2,87) = 8.102, p = .001]. Despite this, including the ‘surprise’ rating (as a proxy for prediction error) as a covariate and modelling an interaction between prediction error, Group and Time did not indicate a significant predictive effect of surprise on any relevant outcomes, so is not reported here.

Figure S1: Dotplot of surprise ratings following binge memory reactivation or non-reactivation on Session 2. 5 = ‘extremely surprised’, -5 ‘completely unsurprised’


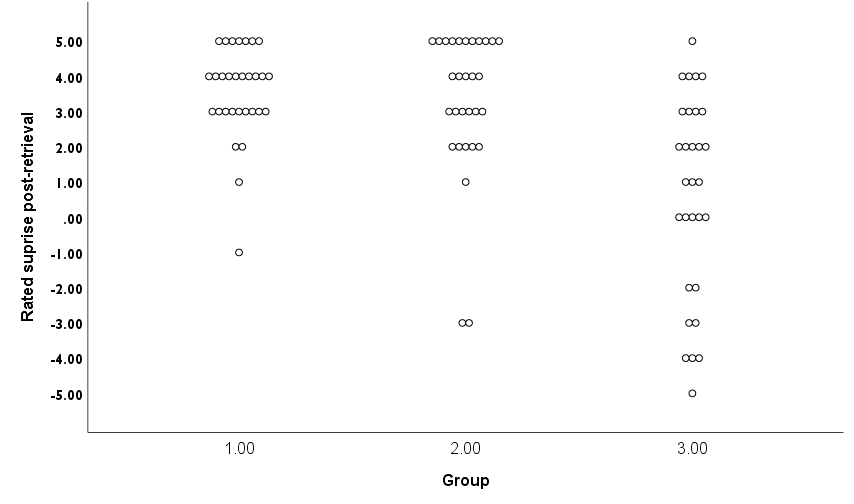


BMR + sham

BMR + RIT

NR + RIT

**RIT task demand compliance**

Overall performance accuracy on the sham and ‘active’ RIT task on Day 2 was very high in all groups, indicating excellent compliance with the Go/No-Go contingency manipulation (see table S2, below). Note that in relative terms. accuracy was significantly lower for binge food images than both LPF images (t(59) = -2.69, p = .026, CI_95%_ = -1.562 to -0.077) and filler images (t(59) = -.2.64, p= .029, CI_95%_ = -1.2 to -0.05), although still extremely high. Inferential tests on signal detection measures could not be computed due to the impossibility of ‘False Alarms’ to LPF stimuli and ‘Misses’ to binge stimuli in the RIT groups (i.e. there were no LPF no-go trials). However false alarm rate to binge images in the RIT groups was very low and almost indiscriminable between the two groups [mean in both groups = 1.4 (t(58) = <.001, p > .999, CI_95%_ = -0.941 to 0.941)]. Misses to LPF stimuli were lower still and did not differ between groups (t(58) = .912, p = .365, CI_95%_ = -1.597 to 0.597)]. In BMR+sham, miss rates were again very low, but false alarms moderately higher for filler image trials than LPF trials, although this did not reach significance (Wilcoxon Z = 1.547, p =.122) and performance was virtually at ceiling across stimuli (Table S3, below).

Table S2: Overall performance accuracy during the RIT or sham procedure.

| Group | Stimulus Type | | Mean±SD | 95% Confidence Interval | |
| --- | --- | --- | --- | --- | --- |
|  |  |  | | Lower | Upper |
| BMR+Sham | Binge | 96.667±0.743 | | 95.19 | 98.143 |
|  | LPF | 98±0.761 | | 96.488 | 99.512 |
|  | Filler | 97.917±0.731 | | 96.463 | 99.371 |
| BMR+RIT | Binge | 98±0.743 | | 96.523 | 99.477 |
|  | LPF | 98.708±0.761 | | 97.196 | 100.221 |
|  | Filler | 98.333±0.731 | | 96.879 | 99.787 |
| NR+RIT | Binge | 97.875±0.743 | | 96.398 | 99.352 |
|  | LPF | 98.292±0.761 | | 96.779 | 99.804 |
|  | Filler | 98.167±0.731 | | 96.713 | 99.621 |

Table S3: Performance metrics in the ‘sham’ training group

|  | Binge | LPF | Filler |
| --- | --- | --- | --- |
| Correct Gos | 39.4±1.163 | 39.3±1.466 | 79.07±1.78 |
| Correct No- Gos | 37.93±4.201 | 39.1±3.642 | 77.6±9.035 |
| Misses | 0.4±1.163 | 0.57±1.455 | 0.73±1.721 |
| False Alarms | 1.7±4.01 | 0.83±3.64 | 2.13±9.066 |

**Sham ‘Taste Test’ Data**

The ratings for dimensions of food taste are each of these are given in table S4, below. Pleasantness was rated -5 (extremely unpleasant) to +5 (most pleasant food ever), due to the bipolar nature of the rating. All other ratings were unipolar and thus rated on a 1-10 scale. Although participants only rated the foods as mildly above neutral pleasantness, they consumed almost all of the food on both study days.

Table S4: Ratings of food consumed during the sham ‘taste test’ following cue reactivity. Values are mean±SD

|  | BMR+Sham | |  | BMR+RIT | |  | NR+RIT | |
| --- | --- | --- | --- | --- | --- | --- | --- | --- |
|  | **Day 1** | **Day 3** |  | **Day 1** | **Day 3** |  | **Day 1** | **Day 3** |
| **Pleasant** | 1.77±1.89 | 1.6±1.73 |  | 1.84±2.42 | 2.11±2.56 |  | 2.1±2.38 | 1.25±1.71 |
| **Sweet** | 6.41±3.35 | 6.33±3.65 |  | 5.48±4.13 | 5.56±3.74 |  | 6.15±3.84 | 5.62±4.26 |
| **Salty** | 2.66±3.48 | 3.1±3.41 |  | 3.74±3.57 | 4.12±3.63 |  | 3.27±3.82 | 3.48±3.78 |
| **Crunchy** | 4.01±3.5 | 4.46±3.44 |  | 3.92±3.88 | 4.26±3.65 |  | 4.14±3.93 | 3.95±3.71 |
| **Greasy** | 3.54±2.92 | 5.01±2.85 |  | 3.81±3.11 | 3.81±3.15 |  | 3.38±3.11 | 4.31±3.26 |
| **leftover weight (g)** | 2.17±6.69 | 2.86±7.5 |  | 1.56±5.38 | 2.42±6.82 |  | 0.79±4.35 | 0.8±3.16 |

**Methodological note on use of ‘Sham’ training control.**

Sham training to control for RIT, as used in the current study, is considered the gold-standard control within this paradigm, although some authors have hypothesised that ‘sham’ itself may constitute active training, dependent upon the degree of participants’ pre-intervention approach bias ^7^. Indeed, we found potentially deleterious effects of ‘sham’ training on signal detection measures of approach bias, indicating that the 50% binge-food approach contingency used in the sham training constituted training of approach responses towards binge food above baseline levels (commensurate with the low levels of observed baseline bias in our sample). Thus, while ‘sham’ training may not be entirely innocuous, it represents a far more realistic comparator for estimating a clinically practicable effect size ^8^. Further, no suggested alternatives to sham training have been empirically tested ^9^ and our findings suggest that even assessment of approach bias may worsen such biases. In spite of these considerations, the changes in approach bias that were observed after training in-lab (whether positive or negative) did not produce any meaningful differences in eating behaviour or eating disorder symptomatology

**REFERENCES:**

1. Blechert, J., Meule, A., Busch, N. A. & Ohla, K. Food-pics: an image database for experimental research on eating and appetite. *Front. Psychol.* **5**, 15037–15042 (2014).

2. Houben, K., Nederkoorn, C., Wiers, R. W. & Jansen, A. Resisting temptation: decreasing alcohol-related affect and drinking behavior by training response inhibition. *Drug Alcohol Depend.* **116**, 132–6 (2011).

3. Houben, K. & Jansen, A. Chocolate equals stop. Chocolate-specific inhibition training reduces chocolate intake and go associations with chocolate. *Appetite* **87**, 318–323 (2015).

4. R Development Core Team. R: A language and environment for statistical computing. R Foundation for Statistical Computing, Vienna, Austria. URL http://www.R-project.org/. *R Found. Stat. Comput. Vienna, Austria.* (2015). doi:10.1007/978-3-540-74686-7

5. Rouder, J. N., Speckman, P. L., Sun, D., Morey, R. D. & Iverson, G. Bayesian t tests for accepting and rejecting the null hypothesis. *Psychon. Bull. Rev.* **16**, 225–237 (2009).

6. Zhang, H. *et al.* A new look at the difference between the GEE and the GLMM when modeling longitudinal count responses. *J. Appl. Stat.* **39**, 2067–2079 (2012).

7. Kakoschke, N., Kemps, E. & Tiggemann, M. What is the appropriate control condition for approach bias modification? A response to commentary by Becker et al. (2017). *Addictive Behaviors* **77**, 295–296 (2018).

8. Becker, D., Jostmann, N. B. & Holland, R. W. Does approach bias modification really work in the eating domain? A commentary on Kakoschke et al. (2017). *Addict. Behav.* **77**, 293–294 (2018).

9. Tiggemann, M. & Kemps, E. Is Sham Training Still Training? An Alternative Control Group for Attentional Bias Modification. *Front. Psychol.* **11**, 15037–15042 (2020).
